# Mol­ecular and crystal structure, lattice energy and DFT calculations of two 2′-(nitro­benzo­yloxy)aceto­phenone isomers

**DOI:** 10.1107/S2056989020006295

**Published:** 2020-05-19

**Authors:** Georgii Bogdanov, Jenna Bustos, Viktor Glebov, Evgenii Oskolkov, John P. Tillotson, Tatiana V. Timofeeva

**Affiliations:** a Department of Chemistry, New Mexico Highlands University, Las Vegas, New Mexico, 87701, USA; b Department of Chemical and Biomolecular Engineering, University of California, Irvine, Irvine, California, 92617, USA; c School of Chemistry and Biochemistry, Georgia Institute of Technology, Atlanta, Georgia, 30332, USA

**Keywords:** 2′-(nitro­benzo­yloxy)aceto­phenone isomers, crystal structure, mol­ecular conformation, DFT calculations, lattice energy

## Abstract

The two isomers 2′-(4-nitro­benzo­yloxy)aceto­phenone (systematic name: 2-acetyl­phenyl 4-nitro­benzoate) (**I**) and 2′-(2-nitro­benzo­yloxy)aceto­phenone (systematic name: 2-acetyl­phenyl 2-nitro­benzoate) (**II**), both C_15_H_11_NO_5_, with *para* and *ortho* positions of the nitro substituent have been crystallized and studied. It is evident that the variation in the position of the nitro group causes a significant difference in the mol­ecular conformations: the dihedral angle between the aromatic fragments in the mol­ecule of **I** is 84.80 (4)*°*, while that in the mol­ecule of **II** is 6.12 (7)*°*. Diffraction analysis revealed the presence of a small amount of water in the crystal of **I**. DFT calculations of the mol­ecular energy demonstrate that the *ortho* substituent causes a higher energy for isomer **II**, while crystal lattice energy calculations show that the values are almost equal for two isomers.

## Abstract 2

The two isomers 2′-(4-nitro­benzo­yloxy)aceto­phenone with *para* and *ortho* positions of the nitro substituent have been crystallized and studied. It is evident that the variation in the position of the nitro group causes a significant difference in the mol­ecular conformations.

## Chemical context   

2′-Benzoyl­oxyaceto­phenones, also known as 2-acetyl­phenyl benzoates, with and without additional substituents are used in the synthesis of materials with different biomedical applications (Singh *et al.*, 2017 ▸; Vyas *et al.*, 2016 ▸; Ali *et al.*, 2017 ▸). The two isomers presented here, 2′-(4-nitro­benzo­yloxy)aceto­phenone (**I**) and 2′-(2-nitro­benzo­yloxy)aceto­phenone (**II**), have been employed as starting materials for the Baker–Venkataraman rearrangement (Baker, 1933 ▸; Mahal & Venkataraman, 1934 ▸) to obtain 1,3-diketones, namely 1-(2-hy­droxy­phen­yl)-3-(nitro­phen­yl)propan-1,3-diones, with different positions of the nitro substituents. These diketones have been used to synthesize substituted nitro­flavones, which are potentially useful as pharmaceutical materials (Barros & Silva, 2006 ▸). Recently, halogen- and/or nitro-substituted phenyl benzoates were found to be plastic crystals. This characteristic is related to the presence of the flexible –C—CO– synthon in the mol­ecules (Saha & Desiraju, 2017 ▸; Saha *et al.*, 2018 ▸).
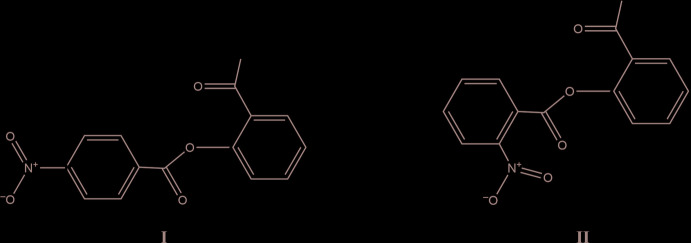


## Structural commentary   

The corresponding bond lengths and bond angles in isomers **I** and **II** are very similar in the two mol­ecules and are close to the standard values. The only unexpected value is angle O1—C14—C7, which is 111.42 (10)° (**I**) and 111.15 (9)° (**II**) for steric reasons, which is quite common for a bridging geometry in mol­ecules with the same mol­ecular core, such as phenyl benzoate and fluorinated phenyl benzoates (Dey & Chopra, 2017 ▸).

**Figure 1:**
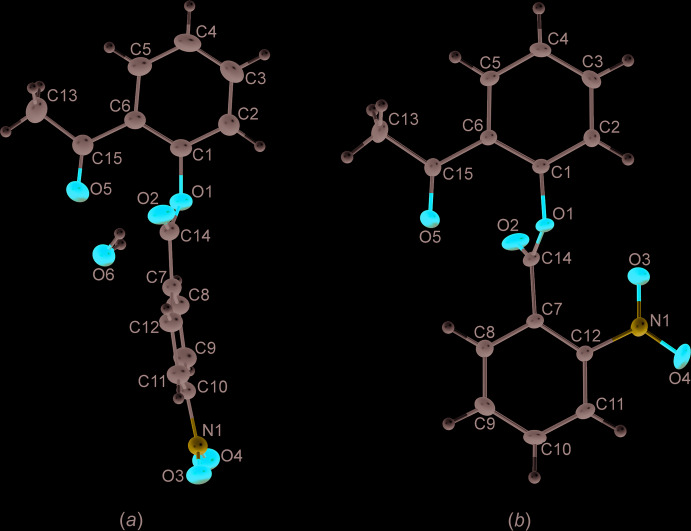
Views of the formula units of (*a*) isomer **I** and (*b*) isomer **II** with the atom-labeling schemes. Displacement ellipsoids are shown with 50% probability. H atoms are shown as fixed spheres of radius 0.15 Å.

In both isomers, the nitro groups lie in the plane of the corresponding phenyl ring [torsion angles C9—C10—N1—O4 = 0.32 (17)° and C7—C12—N1—O3 = 1.08 (14)°, in isomers **I** and **II** respectively], while the acetyl groups are slightly twisted from the phenyl planes [torsion angles C1—C6—C15—O5 = 8.35 (18)° and C1—C6—C15—O5 = 3.97 (16)°]. The conformations of the two mol­ecules are quite different (Fig. 1 ▸). There are two short intra­molecular contacts between the oxygen atoms of the carbonyls and the ether group [O1⋯O5 = 2.694 (1) Å] and the oxygens of two carbonyl groups [O2⋯O5 = 3.008 (2) Å) in mol­ecule **I**; in mol­ecule **II** there are two short contacts between ether oxygen and carbonyl groups [O1⋯O3 and O1⋯O5 = 2.885 (1) and 2.704 (1) Å, respectively].

In the mol­ecule of **I**, the dihedral angle between the phenyl rings is 84.84 (6)° (*i.e.* rings are almost perpendicular to each other), while in the mol­ecule of **II** the phenyl rings are almost parallel, the dihedral angle between them being 6.11 (4)°. It is possible that the significant difference in the mol­ecular conformations of the isomers is caused by the different positions of the nitro substituents.

## DFT calculations   

DFT calculations of isomers **I** and **II** at the B3LYP/6-311G(d,p) level of theory were carried out using *GAUSSIAN 16* software (Frisch *et al.*, 2016 ▸). The geometrical parameters of the two isomers were optimized starting from the mol­ecular geometry in the crystal. No significant differences between the experimental and optimized bond lengths and angles were found. As mentioned above, the observed O1—C14—C7 angles are smaller than the standard value, and the calculated values are also smaller [111.41° (**I**) and 110.04° (**II**), which are very close to experimental values of 111.42 (10)° (**I**) and 111.15 (9)° (**II**)]. A comparison of the conformational characteristics of isomers **I** and **II** according to X-ray data and quantum chemical DFT calculations is presented in Table 1 ▸. This shows that the deviations of the nitro and acetyl groups from the planes of the corresponding aromatic rings are small and almost the same according to the X-ray and DFT data for isomer **I**. The data for isomer **II** indicate that the sterically stressed *ortho* position of the nitro group leads to larger differences between the mol­ecular conformation in the crystal and that calculated for an isolated mol­ecule. Hence, the deviations of the nitro and acetyl groups from the planes of aromatic rings are larger, as well as from the bridging plane, which is different in the isolated mol­ecule of **II**.

## Supra­molecular features   

**Figure 2:**
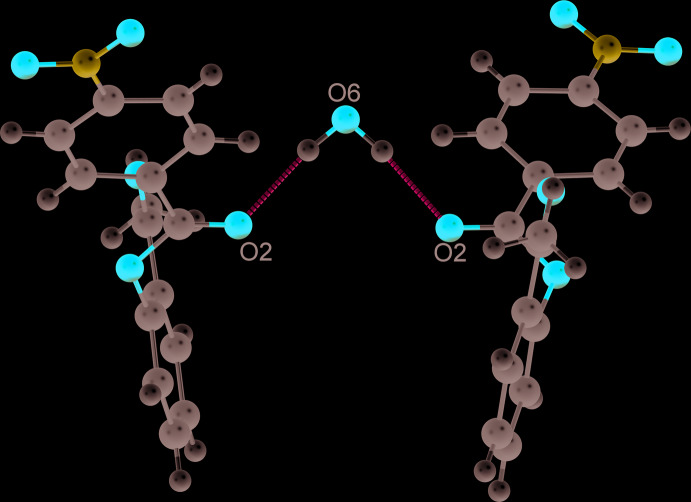
Structure of the dimeric associate in the crystal of **I** with the mol­ecules connected by a 0.074 (2) occupancy bridging water mol­ecule.

**Figure 3:**
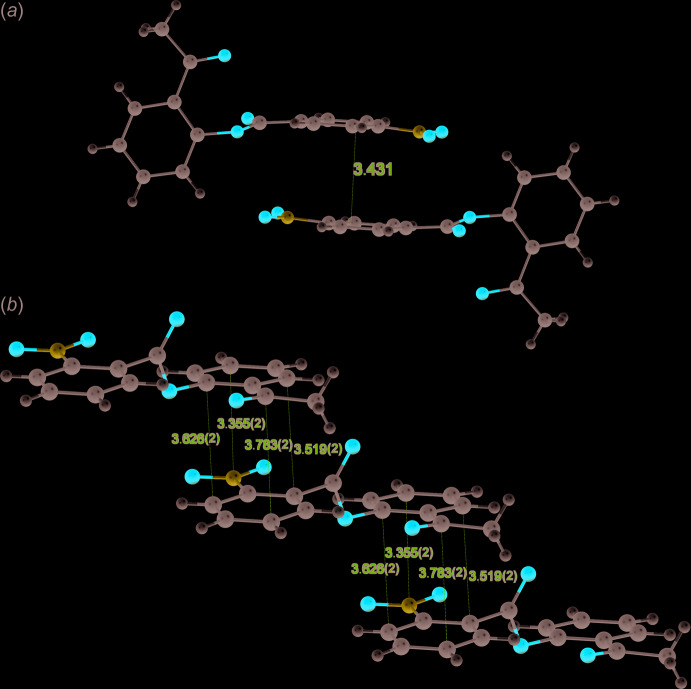
Mol­ecular associates connected by π–π inter­actions in the crystals of **I** (dimer) and **II** (ladder-like chain). In the dimer (**I**), the distance between parallel phenyl rings is given. In the chain (**II**), several short contacts between carbon atoms are indicated.

**Figure 4:**
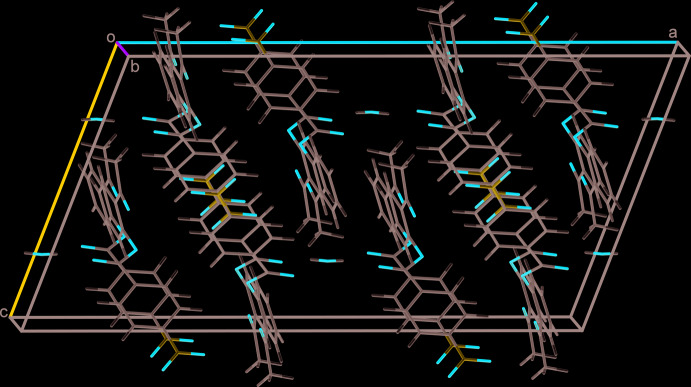
Mol­ecular packing in the crystal of isomer **I**. Mol­ecules of water with 0.074 (2) occupancy are shown.

As a result of the presence in isomers **I** and **II** of oxygen atoms of the carbonyl, nitro, and ether groups, the title mol­ecules are capable of forming C—H⋯O hydrogen bonds (Tables 2 ▸ and 3 ▸). In the crystal of **I**, a low-occupancy [0.074 (2)] partial water mol­ecule forms a bridge between two mol­ecules of **I** (Fig. 2 ▸). The O2⋯O6 distance of 2.912 (6) Å indicates that this inter­action is weak (Brown, 1976 ▸). In addition, π–π inter­actions between phenyl rings are observed in both structures. In **I**, the π–π inter­actions lead to the formation of dimers (Fig. 3 ▸ *a*), while in **II** they lead to the formation of ladder-like chains along the [1 16 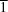] direction (Fig. 3 ▸ *b*). The crystal packing is shown in Figs. 4 ▸ and 5 ▸. Despite the differences in the packing in the crystals of the two isomers, their lattice energies are very similar (see below).

## Lattice energy calculations   

The crystal lattice energies (Table 4 ▸) were calculated using the atom–atom force field implemented in the *CLP-PIXEL* program package (version 3.0, available from http://www.angelogavezzotti.it; Gavezzotti, 2011 ▸). The hydrogen-atom positions for the lattice energy calculations were assigned by the software. In structure **II**, which has a higher packing coefficient, the repulsive and Coulombic components are larger than in the structure of **I**, which has a lower packing coefficient, although the dispersion energy is lower in **I**. The total contribution of all the components results in the lattice energy for the crystals of the two isomers being almost equal. As the amount of water in **I** is low (the water mol­ecule has 0.074 occupancy, see *Refinement* section), it was difficult to evaluate the effect of water on the total lattice energy. However, it is clear that the presence of water makes the structure of **I** less densely packed.

## Database survey   

A search of the Cambridge Crystallographic Database (CSD version 5.40, update of September 2019; Groom *et al.*, 2016 ▸) for the mol­ecules with the same structure as isomers **I** and **II** gave no entries. Three entries were found for the same core structure as in the title mol­ecules. (Adams & Morsi, 1976 ▸ *;* Dey & Chopra, 2017 ▸; Shibakami & Sekiya, 1995 ▸). One is an inclusion compound of phenyl­benzoate with Ni complexes with isonicotinic acid and thio­cyanato coordination bridges (Sekiya *et al.*, 2004 ▸). Several methyl-substituted phenyl­benzoates have been described by Gowda and co-workers, in particular the 2,3-, 2,4- and 2,5-isomers (Gowda, Foro *et al.*, 2008 ▸; Gowda *et al.*, 2009 ▸). Compounds with the same core and nitro-group substituents are rare and are mostly limited to halogen-substituted phenyl­benzoates. The dihedral angles between the two aromatic rings vary. The methyl-substituted compounds tend to have a near-perpendicular geometry with dihedral angles ranging from 73.04 (8) to 87.43 (5)° (Gowda, Tokarcík *et al.*, 2008 ▸; Gowda *et al.*, 2009 ▸), while pure phenyl­benzoate and many of its fluorinated derivatives have angles in the range 52.66 (7) to 62.76 (4)° (Adams & Morsi, 1976 ▸; Dey & Chopra, 2017 ▸). The number of entries in the database for nitro-substituted phenyl­benzoates is limited and is not sufficient for drawing final conclusions on the role of the nitro-group position on the mol­ecular geometry (Saha & Desiraju, 2017 ▸). Finally, the presence of phenyl­benzoate in inclusion compounds seems to have a ‘flattening’ effect on the mol­ecule, lowering the dihedral angle; such a compound was described by Sekiya *et al.* (2004 ▸) with a dihedral angle between the aromatic rings of 20.9 (19)°. Careful analysis of substituted phenyl benzoate derivatives (415 entries in the CSD) presented by Saha & Desiraju (2017 ▸) has shown a strong preference for Ar–Ar torsion angles of between 40 and 90° (91% of entries).

## Synthesis and crystallization   

The synthesis of isomers **I** and **II** was performed according to Barros & Silva (2006 ▸). Crystals of both compounds were grown by slow evaporation from ethanol solution.

## Refinement   

Crystal data, data collection and structure refinement details are summarized in Table 5 ▸. For both structures, the C-bound hydrogen atoms were freely refined. A large residual electron density peak was found for **I**. It was modelled as a partial water mol­ecule. The O6 atom of the water mol­ecule occupies a site on a crystallographic *C*_2_ axis (Fig. 2 ▸). The water mol­ecule was freely refined with a resulting occupation factor of 0.074 (2). The water H atoms were added geometrically taking into account the direction of potential hydrogen bonds in the structure of **I**.

## Supplementary Material

Crystal structure: contains datablock(s) I, II. DOI: 10.1107/S2056989020006295/dj2001sup1.cif

Structure factors: contains datablock(s) I. DOI: 10.1107/S2056989020006295/dj2001Isup2.hkl

Structure factors: contains datablock(s) II. DOI: 10.1107/S2056989020006295/dj2001IIsup3.hkl

Supporting information file. DOI: 10.1107/S2056989020006295/dj2001Isup4.cml

Supporting information file. DOI: 10.1107/S2056989020006295/dj2001IIsup5.cml

CCDC references: 2002786, 2002785

Additional supporting information:  crystallographic information; 3D view; checkCIF report

## Figures and Tables

**Figure 5:**
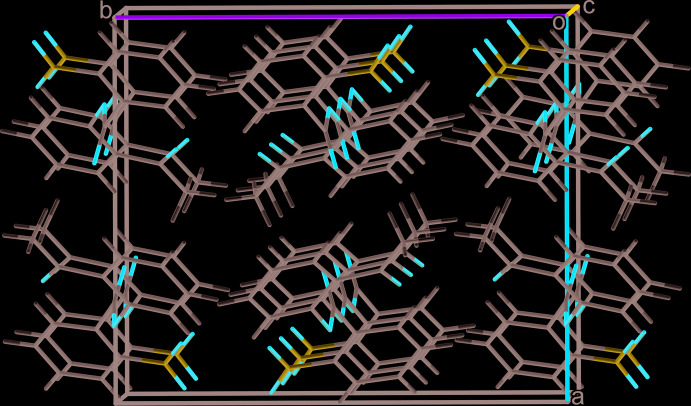
Mol­ecular packing in the crystal of isomer **II**.

**Table 1:** Comparison of conformational characteristics (°) of isomers **I** and **II** from diffraction (X-ray) and computational (DFT) data

Conformational parameters	Isomer **I** – X-ray	Isomer **I** – DFT	Isomer **II** – X-ray	Isomer **II** – DFT
C9—C10—N1—O4	0.32 (17)	0.14	–	–
C11—C12—N1—O4	–	–	−1.10 (15)	−21.06
C5—C6—C15—C13	7.54 (17)	6.03	−3.80 (15)	−10.18
C12—C7—C14—O1	167.74 (11)	172.87	−85.81 (12)	−65.72
C7—C14—O1—C1	−178.76 (10)	171.54	−178.19 (8)	−175.37
C6—C1—O1—C14	−77.59 (14)	−77.23	81.05 (12)	82.74
Ar/Ar	84.80 (4)	87.03	6.12 (7)	21.04

**Table 2:** Hydrogen-bond geometry (Å, °) for **I** [Chem scheme1]

*D*—H⋯*A*	*D*—H	H⋯*A*	*D*⋯*A*	*D*—H⋯*A*
O6—H6⋯O2	0.94 (1)	1.98 (2)	2.912 (6)	173 (2)
C13—H13*A*⋯O2^i^	0.998 (19)	2.638 (19)	3.594 (2)	160.6 (14)
C11—H11⋯O5^ii^	0.960 (16)	2.600 (17)	3.4725 (17)	151.3 (12)

Symmetry codes: (i) 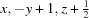; (ii) 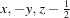.

**Table 3:** Hydrogen-bond geometry (Å, °) for **II** [Chem scheme1]

*D*—H⋯*A*	*D*—H	H⋯*A*	*D*⋯*A*	*D*—H⋯*A*
C11—H11⋯O1^i^	0.941 (16)	2.646 (15)	3.2711 (19)	124.4 (12)

Symmetry code: (i) 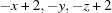.

**Table 4:** Crystal packing characteristics, components of lattice energy and total lattice energy (kJ mol^−1^)

	**I**	**II**
Cell volume, Å	2694.5 (10)	1294.2 (9)
Density, g cm^−3^	1.406	1.464
Packing coefficient	0.739	0.771
Coulombic	−29.9	−35.5
Polarization	−40.2	−40.2
Dispersion	−147.4	−144.1
Repulsion	64.8	67.4
Total	−152.6	−152.3

**Table 5:** Experimental details

	**I**	**II**
Crystal data		
Chemical formula	C_15_H_11_NO_5_·0.07H_2_O	C_15_H_11_NO_5_
*M*_r_	286.58	285.25
Crystal system, space group	Monoclinic, *C*2/*c*	Monoclinic, *P*2_1_/*c*
Temperature (K)	150	100
*a*, *b*, *c* (Å)	26.225 (6), 7.9955 (17), 13.772 (3)	12.209 (5), 14.307 (6), 7.418 (3)
β (°)	111.080 (3)	92.815 (7)
*V* (Å^3^)	2694.5 (10)	1294.2 (9)
*Z*	8	4
Radiation type	Mo *K*α	Mo *K*α
μ (mm^−1^)	0.11	0.11
Crystal size (mm)	0.21 × 0.18 × 0.12	0.15 × 0.15 × 0.1

Data collection		
Diffractometer	Bruker APEXII CCD	Bruker APEXII CCD
Absorption correction	Multi-scan (*SADABS*; Bruker, 2016 ▸)	Multi-scan (*SADABS*, Bruker, 2016 ▸)
*T*_min_, *T*_max_	0.687, 0.746	0.650, 0.746
No. of measured, independent and observed [*I* > 2σ(*I*)] reflections	22632, 4010, 2983	5087, 3620, 2913
*R*_int_	0.056	0.016
(sin θ/λ)_max_ (Å^−1^)	0.711	0.741

Refinement		
*R*[*F* ^2^ > 2σ(*F* ^2^)], *wR*(*F* ^2^), *S*	0.042, 0.116, 1.05	0.040, 0.122, 1.06
No. of reflections	4010	3620
No. of parameters	244	234
No. of restraints	4	0
H-atom treatment	Only H-atom coordinates refined	All H-atom parameters refined
Δρ_max_, Δρ_min_ (e Å^−3^)	0.28, −0.24	0.43, −0.23

Computer programs: *APEX3* and *SAINT* (Bruker, 2016 ▸), *SHELXT2017/1* (Sheldrick, 2015*a* ▸), *SHELXL2017/1* (Sheldrick, 2015*b* ▸), *OLEX2* (Dolomanov *et al.*, 2009 ▸) and *Mercury* (Macrae *et al.*, 2020 ▸).

## supplementary crystallographic information

### 2-Acetylphenyl 4-nitrobenzoate 0.07-hydrate (I) Crystal data

**Table:**

C_15_H_11_NO_5_·0.07H_2_O	*F*(000) = 1190
*M**_r_* = 286.58	*D*_x_ = 1.413 Mg m^−^^3^
Monoclinic, *C*2/*c*	Mo *K*α radiation, λ = 0.71073 Å
*a* = 26.225 (6) Å	Cell parameters from 5275 reflections
*b* = 7.9955 (17) Å	θ = 2.7–29.6°
*c* = 13.772 (3) Å	µ = 0.11 mm^−^^1^
β = 111.080 (3)°	*T* = 150 K
*V* = 2694.5 (10) Å^3^	Block, yellow
*Z* = 8	0.21 × 0.18 × 0.12 mm

### 2-Acetylphenyl 4-nitrobenzoate 0.07-hydrate (I) Data collection

**Table:**

Bruker APEXII CCD diffractometer	2983 reflections with *I* > 2σ(*I*)
φ and ω scans	*R*_int_ = 0.056
Absorption correction: multi-scan (SADABS; Bruker, 2016)	θ_max_ = 30.4°, θ_min_ = 2.7°
*T*_min_ = 0.687, *T*_max_ = 0.746	*h* = −36→37
22632 measured reflections	*k* = −11→11
4010 independent reflections	*l* = −19→19

### 2-Acetylphenyl 4-nitrobenzoate 0.07-hydrate (I) Refinement

**Table:**

Refinement on *F*^2^	Primary atom site location: dual
Least-squares matrix: full	Secondary atom site location: difference Fourier map
*R*[*F*^2^ > 2σ(*F*^2^)] = 0.042	Hydrogen site location: inferred from neighbouring sites
*wR*(*F*^2^) = 0.116	Only H-atom coordinates refined
*S* = 1.05	*w* = 1/[σ^2^(*F*_o_^2^) + (0.0487*P*)^2^ + 1.2105*P*] where *P* = (*F*_o_^2^ + 2*F*_c_^2^)/3
4010 reflections	(Δ/σ)_max_ < 0.001
244 parameters	Δρ_max_ = 0.28 e Å^−^^3^
4 restraints	Δρ_min_ = −0.24 e Å^−^^3^

### 2-Acetylphenyl 4-nitrobenzoate 0.07-hydrate (I) Special details

**Table:**

Geometry. All esds (except the esd in the dihedral angle between two l.s. planes) are estimated using the full covariance matrix. The cell esds are taken into account individually in the estimation of esds in distances, angles and torsion angles; correlations between esds in cell parameters are only used when they are defined by crystal symmetry. An approximate (isotropic) treatment of cell esds is used for estimating esds involving l.s. planes.

### 2-Acetylphenyl 4-nitrobenzoate 0.07-hydrate (I) Fractional atomic coordinates and isotropic or equivalent isotropic displacement parameters (Å^2^)

**Table:**

	*x*	*y*	*z*	*U*_iso_*/*U*_eq_	Occ. (<1)
O1	0.35357 (3)	0.44264 (11)	0.29788 (7)	0.0308 (2)	
O2	0.42547 (3)	0.33442 (12)	0.26638 (7)	0.0371 (2)	
O5	0.40200 (4)	0.22975 (12)	0.45589 (8)	0.0418 (2)	
O3	0.24003 (4)	−0.15624 (13)	−0.10893 (8)	0.0420 (2)	
O4	0.17571 (4)	−0.07242 (14)	−0.05770 (8)	0.0455 (3)	
N1	0.22348 (4)	−0.07748 (13)	−0.04993 (8)	0.0323 (2)	
C6	0.42127 (4)	0.52092 (15)	0.46672 (9)	0.0271 (2)	
C7	0.33704 (5)	0.21458 (15)	0.18295 (9)	0.0266 (2)	
C1	0.38789 (5)	0.56226 (15)	0.36503 (9)	0.0280 (2)	
C14	0.37775 (5)	0.33207 (15)	0.25404 (9)	0.0275 (2)	
C10	0.26387 (5)	0.01845 (14)	0.03499 (9)	0.0281 (2)	
C12	0.35454 (5)	0.11618 (16)	0.11749 (10)	0.0321 (3)	
C15	0.42406 (5)	0.34790 (16)	0.51033 (10)	0.0304 (3)	
C9	0.24572 (5)	0.10844 (16)	0.10176 (10)	0.0325 (3)	
C5	0.45180 (5)	0.65194 (17)	0.52810 (10)	0.0327 (3)	
C11	0.31776 (5)	0.01690 (16)	0.04198 (10)	0.0320 (3)	
C8	0.28291 (5)	0.20799 (16)	0.17673 (10)	0.0313 (3)	
C4	0.44849 (6)	0.81349 (17)	0.48991 (11)	0.0382 (3)	
C2	0.38375 (6)	0.72310 (17)	0.32635 (11)	0.0355 (3)	
C3	0.41424 (6)	0.84948 (17)	0.38984 (12)	0.0397 (3)	
C13	0.45479 (7)	0.3244 (2)	0.62472 (11)	0.0419 (3)	
O6	0.500000	0.0754 (11)	0.250000	0.040 (2)	0.148 (2)
H6	0.474 (2)	0.151 (3)	0.256 (8)	0.048*	0.148 (2)
H5	0.4747 (7)	0.626 (2)	0.5957 (13)	0.040 (4)*	
H12	0.3931 (6)	0.1215 (19)	0.1236 (12)	0.038 (4)*	
H13A	0.4407 (7)	0.398 (2)	0.6677 (14)	0.054 (5)*	
H2	0.3589 (7)	0.745 (2)	0.2553 (14)	0.047 (4)*	
H3	0.4122 (7)	0.961 (2)	0.3652 (13)	0.051 (5)*	
H11	0.3295 (6)	−0.048 (2)	−0.0046 (12)	0.045 (4)*	
H13B	0.4515 (7)	0.208 (2)	0.6428 (14)	0.057 (5)*	
H9	0.2081 (7)	0.1025 (19)	0.0956 (12)	0.040 (4)*	
H4	0.4692 (7)	0.901 (2)	0.5340 (13)	0.052 (5)*	
H8	0.2717 (6)	0.273 (2)	0.2253 (12)	0.041 (4)*	
H13C	0.4953 (9)	0.353 (3)	0.6436 (16)	0.074 (6)*	

### 2-Acetylphenyl 4-nitrobenzoate 0.07-hydrate (I) Atomic displacement parameters (Å^2^)

**Table:**

	*U*^11^	*U*^22^	*U*^33^	*U*^12^	*U*^13^	*U*^23^
O1	0.0267 (4)	0.0308 (4)	0.0320 (4)	−0.0018 (3)	0.0072 (3)	−0.0082 (4)
O2	0.0277 (4)	0.0466 (6)	0.0372 (5)	−0.0066 (4)	0.0119 (4)	−0.0124 (4)
O5	0.0580 (6)	0.0288 (5)	0.0387 (5)	−0.0050 (4)	0.0177 (5)	−0.0028 (4)
O3	0.0455 (5)	0.0399 (5)	0.0357 (5)	−0.0030 (4)	0.0085 (4)	−0.0104 (4)
O4	0.0296 (5)	0.0521 (6)	0.0491 (6)	−0.0116 (4)	0.0072 (4)	−0.0051 (5)
N1	0.0325 (5)	0.0279 (5)	0.0311 (5)	−0.0047 (4)	0.0050 (4)	0.0009 (4)
C6	0.0260 (5)	0.0281 (6)	0.0286 (6)	−0.0013 (4)	0.0116 (4)	−0.0052 (5)
C7	0.0277 (5)	0.0264 (5)	0.0256 (5)	−0.0025 (4)	0.0096 (4)	−0.0011 (4)
C1	0.0262 (5)	0.0282 (6)	0.0297 (6)	−0.0021 (4)	0.0101 (4)	−0.0067 (5)
C14	0.0285 (5)	0.0289 (6)	0.0243 (5)	−0.0018 (4)	0.0088 (4)	−0.0023 (4)
C10	0.0276 (5)	0.0243 (5)	0.0292 (6)	−0.0046 (4)	0.0064 (4)	−0.0012 (4)
C12	0.0261 (5)	0.0355 (6)	0.0358 (6)	−0.0024 (5)	0.0127 (5)	−0.0067 (5)
C15	0.0327 (6)	0.0312 (6)	0.0304 (6)	0.0023 (5)	0.0152 (5)	−0.0021 (5)
C9	0.0263 (6)	0.0346 (6)	0.0386 (7)	−0.0053 (5)	0.0139 (5)	−0.0032 (5)
C5	0.0312 (6)	0.0370 (7)	0.0309 (6)	−0.0041 (5)	0.0124 (5)	−0.0086 (5)
C11	0.0312 (6)	0.0316 (6)	0.0335 (6)	−0.0012 (5)	0.0120 (5)	−0.0070 (5)
C8	0.0303 (6)	0.0333 (6)	0.0336 (6)	−0.0038 (5)	0.0153 (5)	−0.0063 (5)
C4	0.0428 (7)	0.0335 (7)	0.0429 (8)	−0.0110 (6)	0.0212 (6)	−0.0148 (6)
C2	0.0390 (7)	0.0313 (6)	0.0354 (7)	0.0028 (5)	0.0125 (6)	−0.0002 (5)
C3	0.0508 (8)	0.0262 (6)	0.0471 (8)	−0.0023 (6)	0.0238 (7)	−0.0027 (6)
C13	0.0488 (8)	0.0444 (8)	0.0318 (7)	0.0086 (6)	0.0138 (6)	0.0034 (6)
O6	0.037 (5)	0.033 (5)	0.044 (5)	0.000	0.008 (4)	0.000

### 2-Acetylphenyl 4-nitrobenzoate 0.07-hydrate (I) Geometric parameters (Å, º)

**Table:**

O1—C1	1.4076 (14)	C15—C13	1.5010 (19)
O1—C14	1.3503 (14)	C9—C8	1.3879 (17)
O2—C14	1.2011 (14)	C9—H9	0.960 (16)
O5—C15	1.2154 (15)	C5—C4	1.386 (2)
O3—N1	1.2242 (15)	C5—H5	0.932 (17)
O4—N1	1.2192 (14)	C11—H11	0.960 (16)
N1—C10	1.4788 (15)	C8—H8	0.971 (16)
C6—C1	1.3981 (17)	C4—C3	1.378 (2)
C6—C15	1.4994 (18)	C4—H4	0.958 (18)
C6—C5	1.4022 (17)	C2—C3	1.3865 (19)
C7—C14	1.4928 (16)	C2—H2	0.976 (17)
C7—C12	1.3928 (17)	C3—H3	0.945 (18)
C7—C8	1.3922 (16)	C13—H13A	0.998 (19)
C1—C2	1.3813 (18)	C13—H13B	0.976 (19)
C10—C9	1.3801 (18)	C13—H13C	1.02 (2)
C10—C11	1.3818 (17)	O6—H6	0.941 (14)
C12—C11	1.3857 (17)	O6—H6^i^	0.941 (14)
C12—H12	0.985 (15)		
			
C14—O1—C1	116.52 (9)	C8—C9—H9	121.2 (9)
O3—N1—C10	117.89 (10)	C6—C5—H5	117.4 (10)
O4—N1—O3	123.86 (11)	C4—C5—C6	121.56 (13)
O4—N1—C10	118.25 (11)	C4—C5—H5	121.1 (10)
C1—C6—C15	122.83 (10)	C10—C11—C12	117.74 (11)
C1—C6—C5	116.31 (11)	C10—C11—H11	121.6 (9)
C5—C6—C15	120.85 (11)	C12—C11—H11	120.7 (9)
C12—C7—C14	117.11 (10)	C7—C8—H8	119.4 (9)
C8—C7—C14	122.48 (11)	C9—C8—C7	119.71 (11)
C8—C7—C12	120.32 (11)	C9—C8—H8	120.9 (9)
C6—C1—O1	121.30 (11)	C5—C4—H4	119.9 (10)
C2—C1—O1	115.86 (11)	C3—C4—C5	120.25 (12)
C2—C1—C6	122.72 (11)	C3—C4—H4	119.9 (10)
O1—C14—C7	111.42 (10)	C1—C2—C3	119.19 (13)
O2—C14—O1	124.01 (11)	C1—C2—H2	119.0 (10)
O2—C14—C7	124.44 (11)	C3—C2—H2	121.8 (10)
C9—C10—N1	118.48 (10)	C4—C3—C2	119.95 (13)
C9—C10—C11	123.20 (11)	C4—C3—H3	119.2 (10)
C11—C10—N1	118.30 (11)	C2—C3—H3	120.9 (10)
C7—C12—H12	119.1 (9)	C15—C13—H13A	112.0 (10)
C11—C12—C7	120.47 (11)	C15—C13—H13B	108.9 (11)
C11—C12—H12	120.4 (9)	C15—C13—H13C	110.8 (11)
O5—C15—C6	121.75 (11)	H13A—C13—H13B	109.1 (15)
O5—C15—C13	120.46 (12)	H13A—C13—H13C	106.8 (16)
C6—C15—C13	117.79 (11)	H13B—C13—H13C	109.1 (15)
C10—C9—C8	118.44 (11)	H6—O6—H6^i^	99 (3)
C10—C9—H9	120.3 (9)		
			
O1—C1—C2—C3	176.78 (11)	C14—C7—C8—C9	173.48 (12)
O3—N1—C10—C9	179.69 (11)	C10—C9—C8—C7	−0.10 (19)
O3—N1—C10—C11	1.29 (17)	C12—C7—C14—O1	167.74 (11)
O4—N1—C10—C9	0.32 (17)	C12—C7—C14—O2	−8.35 (19)
O4—N1—C10—C11	−178.08 (12)	C12—C7—C8—C9	−2.88 (19)
N1—C10—C9—C8	−175.42 (11)	C15—C6—C1—O1	1.58 (17)
N1—C10—C11—C12	175.74 (11)	C15—C6—C1—C2	177.36 (12)
C6—C1—C2—C3	0.8 (2)	C15—C6—C5—C4	−178.13 (12)
C6—C5—C4—C3	0.7 (2)	C9—C10—C11—C12	−2.58 (19)
C7—C12—C11—C10	−0.5 (2)	C5—C6—C1—O1	−177.11 (10)
C1—O1—C14—O2	−2.64 (17)	C5—C6—C1—C2	−1.33 (18)
C1—O1—C14—C7	−178.76 (10)	C5—C6—C15—O5	−173.02 (12)
C1—C6—C15—O5	8.35 (18)	C5—C6—C15—C13	7.54 (17)
C1—C6—C15—C13	−171.09 (12)	C5—C4—C3—C2	−1.3 (2)
C1—C6—C5—C4	0.58 (18)	C11—C10—C9—C8	2.9 (2)
C1—C2—C3—C4	0.5 (2)	C8—C7—C14—O1	−8.72 (16)
C14—O1—C1—C6	−77.59 (14)	C8—C7—C14—O2	175.18 (12)
C14—O1—C1—C2	106.36 (13)	C8—C7—C12—C11	3.2 (2)
C14—C7—C12—C11	−173.34 (12)		

Symmetry code: (i) −*x*+1, *y*, −*z*+1/2.

### 2-Acetylphenyl 4-nitrobenzoate 0.07-hydrate (I) Hydrogen-bond geometry (Å, º)

**Table:**

*D*—H···*A*	*D*—H	H···*A*	*D*···*A*	*D*—H···*A*
O6—H6···O2	0.94 (1)	1.98 (2)	2.912 (6)	173 (2)
C13—H13*A*···O2^ii^	0.998 (19)	2.638 (19)	3.594 (2)	160.6 (14)
C11—H11···O5^iii^	0.960 (16)	2.600 (17)	3.4725 (17)	151.3 (12)

Symmetry codes: (ii) *x*, −*y*+1, *z*+1/2; (iii) *x*, −*y*, *z*−1/2.

### 2-Acetylphenyl 2-nitrobenzoate (II) Crystal data

**Table:**

C_15_H_11_NO_5_	*F*(000) = 592
*M**_r_* = 285.25	*D*_x_ = 1.464 Mg m^−^^3^
Monoclinic, *P*2_1_/*c*	Mo *K*α radiation, λ = 0.71073 Å
*a* = 12.209 (5) Å	Cell parameters from 2439 reflections
*b* = 14.307 (6) Å	θ = 2.2–31.6°
*c* = 7.418 (3) Å	µ = 0.11 mm^−^^1^
β = 92.815 (7)°	*T* = 100 K
*V* = 1294.2 (9) Å^3^	Prism, yellow
*Z* = 4	0.15 × 0.15 × 0.1 mm

### 2-Acetylphenyl 2-nitrobenzoate (II) Data collection

**Table:**

Bruker APEXII CCD diffractometer	2913 reflections with *I* > 2σ(*I*)
φ and ω scans	*R*_int_ = 0.016
Absorption correction: multi-scan (SADABS, Bruker, 2016)	θ_max_ = 31.8°, θ_min_ = 1.7°
*T*_min_ = 0.650, *T*_max_ = 0.746	*h* = −17→16
5087 measured reflections	*k* = −20→14
3620 independent reflections	*l* = −3→10

### 2-Acetylphenyl 2-nitrobenzoate (II) Refinement

**Table:**

Refinement on *F*^2^	Primary atom site location: dual
Least-squares matrix: full	Secondary atom site location: difference Fourier map
*R*[*F*^2^ > 2σ(*F*^2^)] = 0.040	Hydrogen site location: difference Fourier map
*wR*(*F*^2^) = 0.122	All H-atom parameters refined
*S* = 1.06	*w* = 1/[σ^2^(*F*_o_^2^) + (0.0675*P*)^2^ + 0.172*P*] where *P* = (*F*_o_^2^ + 2*F*_c_^2^)/3
3620 reflections	(Δ/σ)_max_ < 0.001
234 parameters	Δρ_max_ = 0.43 e Å^−^^3^
0 restraints	Δρ_min_ = −0.23 e Å^−^^3^

### 2-Acetylphenyl 2-nitrobenzoate (II) Special details

**Table:**

Geometry. All esds (except the esd in the dihedral angle between two l.s. planes) are estimated using the full covariance matrix. The cell esds are taken into account individually in the estimation of esds in distances, angles and torsion angles; correlations between esds in cell parameters are only used when they are defined by crystal symmetry. An approximate (isotropic) treatment of cell esds is used for estimating esds involving l.s. planes.

### 2-Acetylphenyl 2-nitrobenzoate (II) Fractional atomic coordinates and isotropic or equivalent isotropic displacement parameters (Å^2^)

**Table:**

	*x*	*y*	*z*	*U*_iso_*/*U*_eq_	
C6	0.65734 (8)	0.01262 (7)	0.45205 (14)	0.0158 (2)	
O1	0.79222 (6)	−0.00491 (5)	0.70582 (10)	0.01756 (18)	
O3	0.84475 (8)	−0.14564 (6)	0.97495 (13)	0.0314 (2)	
C5	0.60515 (9)	−0.03010 (8)	0.30108 (16)	0.0193 (2)	
O5	0.67276 (8)	0.15066 (6)	0.63086 (13)	0.0319 (2)	
C12	0.88232 (8)	−0.00579 (7)	1.12279 (15)	0.0166 (2)	
N1	0.89918 (7)	−0.10690 (6)	1.09597 (14)	0.0207 (2)	
C7	0.80754 (8)	0.04285 (7)	1.00989 (15)	0.0164 (2)	
C8	0.79381 (9)	0.13811 (7)	1.04287 (17)	0.0214 (2)	
C1	0.73215 (8)	−0.04200 (7)	0.55600 (14)	0.0160 (2)	
O2	0.64412 (7)	−0.02330 (6)	0.87287 (12)	0.0282 (2)	
C2	0.75414 (9)	−0.13411 (7)	0.51264 (16)	0.0201 (2)	
C14	0.73772 (8)	−0.00042 (7)	0.86012 (15)	0.0174 (2)	
C11	0.94230 (9)	0.03733 (8)	1.26288 (16)	0.0212 (2)	
C3	0.69989 (10)	−0.17469 (8)	0.36297 (17)	0.0237 (2)	
O4	0.96613 (8)	−0.14724 (7)	1.19635 (15)	0.0395 (3)	
C15	0.63287 (9)	0.11279 (7)	0.49603 (16)	0.0191 (2)	
C4	0.62600 (10)	−0.12258 (8)	0.25762 (16)	0.0226 (2)	
C13	0.55711 (10)	0.16657 (8)	0.36771 (19)	0.0268 (3)	
C10	0.92771 (10)	0.13212 (9)	1.29225 (18)	0.0258 (3)	
C9	0.85407 (10)	0.18239 (8)	1.18253 (18)	0.0255 (3)	
H2	0.8073 (13)	−0.1705 (11)	0.587 (2)	0.035 (4)*	
H8	0.7406 (12)	0.1752 (10)	0.968 (2)	0.028 (4)*	
H11	0.9935 (13)	0.0017 (11)	1.333 (2)	0.032 (4)*	
H5	0.5541 (13)	0.0044 (10)	0.226 (2)	0.031 (4)*	
H13A	0.4825 (13)	0.1366 (11)	0.359 (2)	0.040 (4)*	
H10	0.9702 (14)	0.1641 (12)	1.387 (2)	0.043 (5)*	
H4	0.5886 (13)	−0.1483 (11)	0.149 (2)	0.039 (4)*	
H3	0.7153 (12)	−0.2407 (11)	0.337 (2)	0.033 (4)*	
H9	0.8436 (13)	0.2498 (12)	1.199 (2)	0.041 (4)*	
H13B	0.5874 (14)	0.1732 (12)	0.248 (2)	0.048 (5)*	
H13C	0.5493 (16)	0.2303 (14)	0.415 (3)	0.061 (6)*	

### 2-Acetylphenyl 2-nitrobenzoate (II) Atomic displacement parameters (Å^2^)

**Table:**

	*U*^11^	*U*^22^	*U*^33^	*U*^12^	*U*^13^	*U*^23^
C6	0.0176 (4)	0.0157 (4)	0.0141 (5)	−0.0014 (3)	−0.0002 (4)	0.0012 (3)
O1	0.0176 (3)	0.0212 (4)	0.0135 (4)	−0.0011 (3)	−0.0029 (3)	−0.0015 (3)
O3	0.0479 (5)	0.0180 (4)	0.0272 (5)	−0.0031 (4)	−0.0111 (4)	−0.0016 (3)
C5	0.0222 (5)	0.0208 (5)	0.0145 (6)	−0.0019 (4)	−0.0033 (4)	0.0015 (4)
O5	0.0454 (5)	0.0182 (4)	0.0307 (6)	0.0037 (4)	−0.0132 (4)	−0.0063 (3)
C12	0.0171 (4)	0.0169 (5)	0.0155 (5)	0.0000 (3)	−0.0006 (4)	0.0006 (4)
N1	0.0220 (4)	0.0186 (4)	0.0213 (5)	0.0013 (3)	−0.0006 (4)	0.0030 (3)
C7	0.0178 (4)	0.0180 (5)	0.0133 (5)	−0.0013 (3)	−0.0016 (4)	0.0003 (3)
C8	0.0252 (5)	0.0185 (5)	0.0201 (6)	0.0016 (4)	−0.0030 (4)	0.0003 (4)
C1	0.0171 (4)	0.0178 (5)	0.0129 (5)	−0.0010 (3)	−0.0003 (4)	−0.0004 (4)
O2	0.0227 (4)	0.0441 (5)	0.0177 (5)	−0.0094 (3)	−0.0019 (3)	−0.0001 (4)
C2	0.0243 (5)	0.0173 (5)	0.0186 (6)	0.0030 (4)	−0.0006 (4)	0.0010 (4)
C14	0.0197 (5)	0.0180 (5)	0.0141 (6)	0.0003 (4)	−0.0040 (4)	0.0016 (4)
C11	0.0195 (5)	0.0262 (5)	0.0174 (6)	0.0004 (4)	−0.0048 (4)	−0.0003 (4)
C3	0.0340 (6)	0.0169 (5)	0.0202 (6)	0.0003 (4)	0.0019 (5)	−0.0032 (4)
O4	0.0411 (5)	0.0269 (5)	0.0484 (7)	0.0120 (4)	−0.0185 (5)	0.0045 (4)
C15	0.0210 (5)	0.0153 (5)	0.0207 (6)	−0.0008 (4)	−0.0006 (4)	0.0024 (4)
C4	0.0311 (5)	0.0211 (5)	0.0152 (6)	−0.0044 (4)	−0.0029 (4)	−0.0029 (4)
C13	0.0288 (6)	0.0190 (5)	0.0319 (7)	0.0034 (4)	−0.0064 (5)	0.0058 (5)
C10	0.0267 (5)	0.0291 (6)	0.0209 (7)	−0.0042 (4)	−0.0052 (5)	−0.0079 (5)
C9	0.0314 (6)	0.0193 (5)	0.0256 (7)	−0.0013 (4)	−0.0004 (5)	−0.0052 (4)

### 2-Acetylphenyl 2-nitrobenzoate (II) Geometric parameters (Å, º)

**Table:**

C6—C5	1.4014 (16)	C1—C2	1.3860 (15)
C6—C1	1.4036 (15)	O2—C14	1.1969 (14)
C6—C15	1.5031 (15)	C2—C3	1.3914 (17)
O1—C1	1.4053 (13)	C2—H2	0.981 (16)
O1—C14	1.3534 (14)	C11—C10	1.3865 (17)
O3—N1	1.2232 (13)	C11—H11	0.941 (16)
C5—C4	1.3884 (17)	C3—C4	1.3824 (18)
C5—H5	0.954 (15)	C3—H3	0.984 (16)
O5—C15	1.2179 (15)	C15—C13	1.5057 (17)
C12—N1	1.4762 (15)	C4—H4	0.981 (17)
C12—C7	1.3943 (15)	C13—H13A	1.006 (16)
C12—C11	1.3864 (16)	C13—H13B	0.986 (18)
N1—O4	1.2230 (13)	C13—H13C	0.98 (2)
C7—C8	1.3961 (16)	C10—C9	1.3843 (19)
C7—C14	1.5004 (15)	C10—H10	0.969 (17)
C8—C9	1.3932 (17)	C9—H9	0.981 (17)
C8—H8	0.987 (15)		
			
C5—C6—C1	117.02 (9)	O2—C14—O1	124.38 (10)
C5—C6—C15	120.13 (10)	O2—C14—C7	124.35 (10)
C1—C6—C15	122.85 (10)	C12—C11—C10	119.06 (11)
C14—O1—C1	115.32 (8)	C12—C11—H11	118.9 (9)
C6—C5—H5	120.0 (9)	C10—C11—H11	122.0 (9)
C4—C5—C6	121.32 (11)	C2—C3—H3	118.0 (9)
C4—C5—H5	118.7 (9)	C4—C3—C2	119.87 (10)
C7—C12—N1	119.98 (9)	C4—C3—H3	122.2 (9)
C11—C12—N1	117.71 (9)	C6—C15—C13	118.20 (10)
C11—C12—C7	122.30 (10)	O5—C15—C6	121.78 (10)
O3—N1—C12	117.94 (9)	O5—C15—C13	120.02 (10)
O4—N1—O3	123.77 (10)	C5—C4—H4	117.8 (9)
O4—N1—C12	118.29 (10)	C3—C4—C5	120.31 (11)
C12—C7—C8	117.55 (10)	C3—C4—H4	121.9 (9)
C12—C7—C14	124.77 (9)	C15—C13—H13A	110.4 (9)
C8—C7—C14	117.64 (10)	C15—C13—H13B	112.0 (10)
C7—C8—H8	120.5 (8)	C15—C13—H13C	108.5 (11)
C9—C8—C7	120.71 (11)	H13A—C13—H13B	111.2 (14)
C9—C8—H8	118.8 (8)	H13A—C13—H13C	108.4 (15)
C6—C1—O1	121.64 (9)	H13B—C13—H13C	106.1 (15)
C2—C1—C6	122.01 (10)	C11—C10—H10	120.5 (10)
C2—C1—O1	116.30 (9)	C9—C10—C11	120.04 (11)
C1—C2—C3	119.46 (10)	C9—C10—H10	119.5 (10)
C1—C2—H2	120.3 (9)	C8—C9—H9	118.1 (10)
C3—C2—H2	120.3 (9)	C10—C9—C8	120.33 (11)
O1—C14—C7	111.15 (9)	C10—C9—H9	121.5 (10)
			
C6—C5—C4—C3	−0.37 (17)	C8—C7—C14—O2	−79.61 (14)
C6—C1—C2—C3	−0.77 (16)	C1—C6—C5—C4	0.55 (16)
O1—C1—C2—C3	−178.27 (9)	C1—C6—C15—O5	−3.97 (16)
C5—C6—C1—O1	177.39 (9)	C1—C6—C15—C13	175.80 (10)
C5—C6—C1—C2	0.02 (15)	C1—O1—C14—C7	−178.19 (8)
C5—C6—C15—O5	176.43 (10)	C1—O1—C14—O2	−1.95 (15)
C5—C6—C15—C13	−3.80 (15)	C1—C2—C3—C4	0.95 (17)
C12—C7—C8—C9	0.76 (16)	C2—C3—C4—C5	−0.40 (18)
C12—C7—C14—O1	−85.81 (12)	C14—O1—C1—C6	81.05 (12)
C12—C7—C14—O2	97.95 (14)	C14—O1—C1—C2	−101.44 (11)
C12—C11—C10—C9	0.02 (18)	C14—C7—C8—C9	178.50 (10)
N1—C12—C7—C8	179.17 (9)	C11—C12—N1—O3	178.49 (10)
N1—C12—C7—C14	1.61 (15)	C11—C12—N1—O4	−1.10 (15)
N1—C12—C11—C10	−179.57 (10)	C11—C12—C7—C8	−0.38 (15)
C7—C12—N1—O3	−1.08 (14)	C11—C12—C7—C14	−177.94 (10)
C7—C12—N1—O4	179.33 (10)	C11—C10—C9—C8	0.36 (18)
C7—C12—C11—C10	−0.01 (16)	C15—C6—C5—C4	−179.82 (10)
C7—C8—C9—C10	−0.76 (18)	C15—C6—C1—O1	−2.23 (15)
C8—C7—C14—O1	96.63 (11)	C15—C6—C1—C2	−179.60 (10)

### 2-Acetylphenyl 2-nitrobenzoate (II) Hydrogen-bond geometry (Å, º)

**Table:**

*D*—H···*A*	*D*—H	H···*A*	*D*···*A*	*D*—H···*A*
C11—H11···O1^i^	0.941 (16)	2.646 (15)	3.2711 (19)	124.4 (12)

Symmetry code: (i) −*x*+2, −*y*, −*z*+2.

## References

[bb1] Adams, J. M. & Morsi, S. E. (1976). *Acta Cryst.* B**32**, 1345–1347.

[bb2] Ali, N. M., Yeap, S. K., Abu, N., Lim, K. L., Ky, H., Pauzi, A. Z. M., Ho, W. Y., Tan, S. W., Alan-Ong, H. K., Zareen, S., Alitheen, N. B. & Akhtar, M. N. (2017). *Cancer Cell Int.* **17**, 30–42. 10.1186/s12935-017-0400-3 PMC5320730 28239299

[bb3] Baker, W. (1933). *J. Chem. Soc.* 1381–1389.

[bb4] Barros, A. I. R. N. A. & Silva, A. M. S. (2006). *Monatsh. Chem.* **137**, 1505–1528.

[bb5] Brown, I. D. (1976). *Acta Cryst.* A**32**, 24–31.

[bb6] Bruker (2016). *APEX3* and *SAINT*. Bruker AXS Inc., Madison, Wisconsin, USA.

[bb7] Dey, D. & Chopra, D. (2017). *Cryst. Growth Des.* **17**, 5117–5128.

[bb24] Dolomanov, O. V., Bourhis, L. J., Gildea, R. J., Howard, J. A. K. & Puschmann, H. (2009). *J. Appl. Cryst.* **42**, 339–341.

[bb8] Frisch, M. J., Trucks, G. W., Schlegel, H. B., Scuseria, G. E., Robb, M. A., Cheeseman, J. R., Scalmani, G., Barone, V., Mennucci, B., Petersson, G. A., Nakatsuji, H., Caricato, M., Li, X., Hratchian, H. P., Izmaylov, A. F., Bloino, J., Zheng, G., Sonnenberg, J. L., Hada, M., Ehara, M., Toyota, K., Fukuda, R., Hasegawa, J., Ishida, M., Nakajima, T., Honda, Y., Kitao, O., Nakai, H., Vreven, T., Montgomery, J. A., Jr., Peralta, J. E., Ogliaro, F., Bearpark, M., Heyd, J. J., Brothers, E., Kudin, K. N., Staroverov, V. N., Kobayashi, R., Normand, J., Raghavachari, K., Rendell, A., Burant, J. C., Iyengar, S. S., Tomasi, J., Cossi, M., Rega, N., Millam, J. M., Klene, M., Knox, J. E., Cross, J. B., Bakken, V., Adamo, C., Jaramillo, J., Gomperts, R., Stratmann, R. E., Yazyev, O., Austin, A. J., Cammi, R., Pomelli, C., Ochterski, J. W., Martin, R. L., Morokuma, K., Zakrzewski, V. G., Voth, G. A., Salvador, P., Dannenberg, J. J., Dapprich, S., Daniels, A. D., Farkas, Ö., Foresman, J. B., Ortiz, J. V., Cioslowski, J. & Fox, D. J. (2016). *GAUSSIAN09*. Gaussian Inc., Wallingford, CT, USA. http://www.gaussian.com.

[bb9] Gavezzotti, A. (2011). *New J. Chem.* **35**, 1360–1368.

[bb10] Gowda, B. T., Foro, S., Babitha, K. S. & Fuess, H. (2008). *Acta Cryst.* E**64**, o1587. 10.1107/S1600536808022721 PMC2962204 21203285

[bb11] Gowda, B. T., Tokarčík, M., Kožíšek, J., Babitha, K. S. & Fuess, H. (2008). *Acta Cryst.* E**64**, o1280. 10.1107/S1600536808017480 PMC2961695 21202912

[bb12] Gowda, B. T., Tokarčík, M., Kožíšek, J., Suchetan, P. A. & Fuess, H. (2009). *Acta Cryst.* E**65**, o2599. 10.1107/S1600536809039397 PMC2971170 21578218

[bb13] Groom, C. R., Bruno, I. J., Lightfoot, M. P. & Ward, S. C. (2016). *Acta Cryst.* B**72**, 171–179. 10.1107/S2052520616003954 PMC4822653 27048719

[bb23] Macrae, C. F., Sovago, I., Cottrell, S. J., Galek, P. T. A., McCabe, P., Pidcock, E., Platings, M., Shields, G. P., Stevens, J. S., Towler, M. & Wood, P. A. (2020). *J. Appl. Cryst.* **53**, 226–235. 10.1107/S1600576719014092 PMC6998782 32047413

[bb14] Mahal, H. S. & Venkataraman, K. (1934). *J. Chem. Soc.* pp. 1767–1769.

[bb15] Saha, S. & Desiraju, G. R. (2017). *J. Am. Chem. Soc.* **139**, 1975–1983. 10.1021/jacs.6b11835 28080045

[bb16] Saha, S., Mishra, M. K., Reddy, C. M. & Desiraju, G. R. (2018). *Acc. Chem. Res.* **51**, 2957–2967. 10.1021/acs.accounts.8b00425 30351918

[bb17] Sekiya, R., Nishikiori, S.-I. & Ogura, K. (2004). *J. Am. Chem. Soc.* **126**, 16587–16600. 10.1021/ja0463280 15600365

[bb18] Sheldrick, G. M. (2015*a*). *Acta Cryst.* A**71**, 3–8.

[bb19] Sheldrick, G. M. (2015*b*). *Acta Cryst.* C**71**, 3–8.

[bb20] Shibakami, M. & Sekiya, A. (1995). *Acta Cryst.* C**51**, 326–330.

[bb21] Singh, M., Kaur, M., Vyas, B. & Silakari, O. (2017). *J. Med. Chem.* **27**, 520–530.

[bb22] Vyas, B., Singh, M., Kaur, M., Silakari, O., Bahia, M. S. & Singh, B. (2016). *J. Med. Chem.* **25**, 609–626.

